# Cardiac Hydatid Cyst Successfully Managed with Albendazole: A Case Report

**DOI:** 10.7759/cureus.6405

**Published:** 2019-12-17

**Authors:** Reem A AlShamlan, Abdulrahman M Almousa, Mohammed J Al Saeed, Faisal H Al-Dera, Murtadha A Alobaydun

**Affiliations:** 1 Family and Community Medicine, Imam Abdulrahman Bin Faisal University, Dammam, SAU; 2 Radiology, Taibah University, Medina, SAU; 3 Radiology, Imam Abdulrahman Bin Faisal University, Dammam, SAU

**Keywords:** cardiac hydatid cyst, hydatid disease, echinococcus granulosus, computer tomography

## Abstract

Hydatid disease is a parasitic infection caused by tapeworm *Echinococcus*. It has a worldwide distribution, but it is endemic in certain geographic locations. Hydatid disease can almost involve any body organ. However, cardiac involvement is exceedingly rare. We report the case of young girl presenting with chest pain and shortness of breath. She had a history of renal hydatid cyst for which she underwent surgical resection. After thorough investigation, she was found to have a hydatid cyst involving the left ventricular wall. The patient’s family refused surgical management, and she had received medical treatment in the form of albendazole which showed dramatic improvement in her condition. This case shed light on the importance of having a high index of suspicion for this condition, particularly in those patients with a history of hydatid cysts in other organs

## Introduction

Hydatid disease is a zoonotic disease caused by infection with the metacestode stage of the tapeworm *Echinococcus*. The *Echinococcus* species have different geographic distributions and involve different hosts, but the *Echinococcus granulosus* and *Echinococcus multilocularis *are the most frequent, causing cystic echinococcosis and alveolar echinococcosis, respectively [1].

The clinical manifestation of hydatid disease depends on the organs involved and the number and size of the cysts. While hydatid cyst may involve any body organ, the most commonly affected organs are the liver and the lungs, as they are involved in approximately two-thirds and one-fourth of all cases, respectively [2]. Herein, we report the case of girl presenting with chest pain and shortness of breath. After investigation, she was diagnosed as having hydatid cyst involving the left ventricular wall. The patient’s family refused surgical management, and she had received medical treatment which showed dramatic improvement in her condition. Cardiac hydatid disease is very rare, with only a few cases reported in the literature [3].

## Case presentation

We report a case of a 10-year-old girl who presented to the emergency department with a two-week history of progressive shortness of breath and chest pain for which she had not sought medical attention before. She lives in a rural country in Syria. She did not report history of cough, wheezing, or fever. The patient surgical history is remarkable for renal hydatid cyst for which she underwent surgical excision one year prior to her current presentation. She is not taking any medications on a routine basis. She is a student in the primary school and had unremarkable family and social history.

Upon examination, she was afebrile, and her pulse rate, blood pressure, respiratory rate, temperature, and oxygen saturation were 70 beats/min, 100/70 mmHg, 36.9°C, and 99% on room air, respectively. Examination of the precordium revealed normal S1 and S2 heart sounds with no added sounds or murmurs. Auscultation of the chest revealed normal vesicular breath sounds throughout both lung fields with no added sounds. The rest of the physical examination results were also insignificant. In addition, the complete blood count revealed a hemoglobin level of 14.2 g/dL, a leukocyte count of 7.5 × 10^3^/mL, and a platelet count of 462 × 10^3^/mL, with her levels of urea, creatinine, electrolytes, and liver enzymes found to be within the normal ranges.

**Figure 1:**
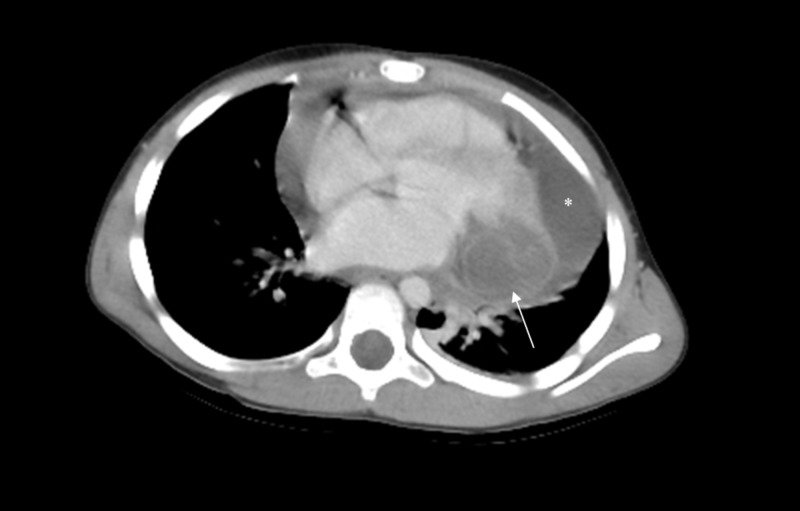
CT of the Heart CT scan of the heart demonstrating pericardial effusion (asterisk) and the presence of cystic lesion involving the left ventricular wall (arrow)

The chest X-ray performed did not reveal any abnormalities. The electrocardiography revealed a normal sinus rhythm. The patient underwent a bedside transthoracic echocardiography which demonstrated a pericardial effusion and an epicardial echolucent lesion in the left ventricle. Subsequently, computed tomography (CT) scan was performed which was suggestive of the presence of a large hydatid cyst in the free wall of the left ventricle (Figure 1). The CT scan did not identify any lung lesions, and abdominal ultrasound was unremarkable. An enzyme-linked immunosorbent assay (ELISA) test was performed and confirmed the presence of anti-*Echinococcus* antibodies.

The patient’s family was counseled regarding the diagnosis of cardiac hydatid cyst and its management. However, the family had strongly disagreed with the plan of carrying out a cardiac operation for excision of this cyst despite providing them with the detailed information about the possible consequences. Hence, the patient was started on albendazole (400 mg) twice daily. The patient had regular follow-up visits in our clinic, and the symptoms showed complete resolution. In the 12-month follow-up visit, the bedside echocardiography revealed a significant reduction in cardiac hydatid cyst as compared to that at the time of initial presentation.

## Discussion

The term hydatid cyst was first coined in 1800 and refers to a “drop of water.” Cardiac hydatid disease, an exceedingly rare condition, was first described by Williams in 1836 [4]. It represents less than 2% of all cases of hydatid disease [3].

Hydatid disease is often asymptomatic, particularly in children. Considering the slow growth of cysts, hydatid disease typically gives clinical signs and symptoms after decades [5]. However, as cardiac location might be associated with significant impairment, the age of presentation is younger than that of the usual hydatid disease [6].

The symptoms of cardiac hydatid disease range from being asymptomatic to having life-threatening course. As in our case, cardiac hydatid disease may present with chest pain and shortness of breath. Additionally, palpitations and recurrent syncope may occur and are related to underlying cardiac arrhythmias. Rupture of cardiac hydatid cyst can result in pulmonary embolism or stroke [7,8]. The release of cyst content induces a life-threatening allergic reaction, which might also be a complication during surgical excision of hydatid cysts [9]. However, some patients may demonstrate infection-associated symptoms such as fever, cough, and fatigue, which make the initial differential diagnosis of cardiac hydatid disease difficult [6].

The most important diagnostic hint for cardiac hydatid disease is the origin of the patient or a history of travel to an endemic region. In our case, reaching the diagnosis was not challenging as the patient lives in an endemic area and had a history of renal hydatid disease.

The cardiac hydatid cyst is most frequently found in the left ventricle, according to the coronary circulation. It can also occur in the right ventricle, left atrium, the septum, and pericardium [10]. Elevation of cardiac enzymes might occur as a result of decreased coronary perfusion due to the effect of the cysts [11]. Atrial natriuretic peptide may also be detectable if the cardiac cyst was associated with heart failure by impairing the relaxation of the ventricles [12]. In our case, however, cardiac enzymes were not elevated. The ELISA test is considered to be a highly sensitive investigation for hydatid disease, and it does correlate with the cyst size making it useful in monitoring the disease progression [13]. Moreover, imaging diagnosis of cardiac hydatid disease is always needed for the management. As in our case, echocardiography and CT scan are useful modalities.

The management of hydatid disease should be based on multidisciplinary approach in a special medical center involving collaboration between surgeons, cardiologists, radiologists, infection disease specialists. In all cases of cardiac hydatid disease, surgical excision is the mainstay of treatment, even in asymptomatic patients because of the risk of cyst rupture [14]. In our case, however, the family refused the surgical management despite the extensive counseling. In such conditions or with inoperable disease, medical treatment with anthelmintic therapy is an alternative option.

## Conclusions

Cardiac hydatid disease is a rare condition. This case shed light on the importance of having a high index of suspicion for this condition, particularly in those patients with a history of hydatid cysts in other organs.
